# An *Ambystoma mexicanum *EST sequencing project: analysis of 17,352 expressed sequence tags from embryonic and regenerating blastema cDNA libraries

**DOI:** 10.1186/gb-2004-5-9-r67

**Published:** 2004-08-13

**Authors:** Bianca Habermann, Anne-Gaelle Bebin, Stephan Herklotz, Michael Volkmer, Kay Eckelt, Kerstin Pehlke, Hans Henning Epperlein, Hans Konrad Schackert, Glenis Wiebe, Elly M Tanaka

**Affiliations:** 1 Scionics Computer Innovation GmbH, Pfotenhauerstrasse 110, Dresden 01307, Germany; 2 Max Planck Institute of Molecular Cell Biology and Genetics, Pfotenhauerstrasse 108, Dresden 01307, Germany; 3 Institute of Anatomy, Medical Faculty of the Carl Gustav Carus Technical University, Dresden, Fetscherstrasse 74, Dresden 01307, Germany; 4 Department of Surgical Research, Medical Faculty of the Carl Gustav Carus Technical University, Dresden, Fetscherstrasse 74, Dresden 01307, Germany

## Abstract

**Background:**

The ambystomatid salamander, *Ambystoma mexicanum *(axolotl), is an important model organism in evolutionary and regeneration research but relatively little sequence information has so far been available. This is a major limitation for molecular studies on caudate development, regeneration and evolution. To address this lack of sequence information we have generated an expressed sequence tag (EST) database for *A. mexicanum*.

**Results:**

Two cDNA libraries, one made from stage 18-22 embryos and the other from day-6 regenerating tail blastemas, generated 17,352 sequences. From the sequenced ESTs, 6,377 contigs were assembled that probably represent 25% of the expressed genes in this organism. Sequence comparison revealed significant homology to entries in the NCBI non-redundant database. Further examination of this gene set revealed the presence of genes involved in important cell and developmental processes, including cell proliferation, cell differentiation and cell-cell communication. On the basis of these data, we have performed phylogenetic analysis of key cell-cycle regulators. Interestingly, while cell-cycle proteins such as the cyclin B family display expected evolutionary relationships, the cyclin-dependent kinase inhibitor 1 gene family shows an unusual evolutionary behavior among the amphibians.

**Conclusions:**

Our analysis reveals the importance of a comprehensive sequence set from a representative of the Caudata and illustrates that the EST sequence database is a rich source of molecular, developmental and regeneration studies. To aid in data mining, the ESTs have been organized into an easily searchable database that is freely available online.

## Abstract 2

An EST database has been generated for the axolotl *Ambystoma mexicanum*. Analysis of this data has uncovered an unusual phylogenetic distribution of the cyclin dependent kinase inhibitor 1 gene family in amphibians.

## Background

The Caudata (tailed amphibians such as salamanders) are a major focus of work in vertebrate evolution and speciation [1,2]. The salamander is also an important vertebrate model organism for understanding regeneration, being one of the few vertebrates that is able to regenerate entire body structures such as the limb, tail and jaw as an adult. Despite the pivotal role of this animal order in research, comparatively little sequence information is available. In contrast, 458,413 nucleotide sequences exist for the Anura (frogs and toads). This high number is primarily attributable to large EST sequencing efforts for the model organisms for embryology - *Xenopus laevis *and *Silurana tropicalis.*

A salamander EST project is particularly important as these organisms have extremely large genomes, making a genome project unwieldy and unlikely without specialized approaches such as methylation filtration [3]. Genome sizes range from 8.5 billion base pairs for *Desmognathus monticola *(seal salamander) to nearly 70 billion base pairs for *Plethodon vandykei *(Van Dyke's salamander) [4]. The ambystomatid *Ambystoma mexicanum*, a species important for studies in evolution, regeneration and development, has an estimated genome size between 21.9 billion and 48 billion base pairs [5,6] and measurements of its genome in centimorgans (cM) has yielded the largest size reported for a living vertebrate so far (7,291 cM [7]). In maize, another organism with a large genome, 60,000 sequence reads were required before genome sequencing of methylation-filtered genomic libraries generated significantly more gene sequence information than the available maize EST sequences [8].

Molecular evolution studies of salamanders have relied primarily on mitochondrial genes such as those for ribosomal RNAs and cytochrome *c *[9]. The lack of sequence information among the Caudata hinders the ability to perform sequence comparison with other important gene families. Furthermore, because of the lack of clones, the number of molecular markers available to study salamander embryology and regeneration is low. To address this gap in sequence availability we have generated a large gene sequence set for *A. mexicanum*. We chose this species because of its role in evolutionary, developmental and regeneration studies. *A. mexicanum *is easily bred in the laboratory, and animals can be obtained from a large, NSF-funded colony [10]. We have sequenced inserts from two cDNA libraries, one produced from dorsal regions of stage 18-22 embryos, consisting primarily of neural tube, somite and notochord. The second library was constructed from day-6 regenerating tail blastema tissue. By sequencing from these two sources, our goal was to obtain sequences of transcripts involved in organizing and regenerating the primary body axis. Here we describe the EST gene set, provide an example of molecular phylogenetic analysis of one gene from this collection, and describe the database created for organizing the *A. mexicanum *EST information. This database is also being implemented for EST sequences from a full-length *X. laevis *cDNA library, and for sequences from a *Canis familiaris *EST project.

## Results

### Assessment of library and EST sequence quality

**Figure 1:**
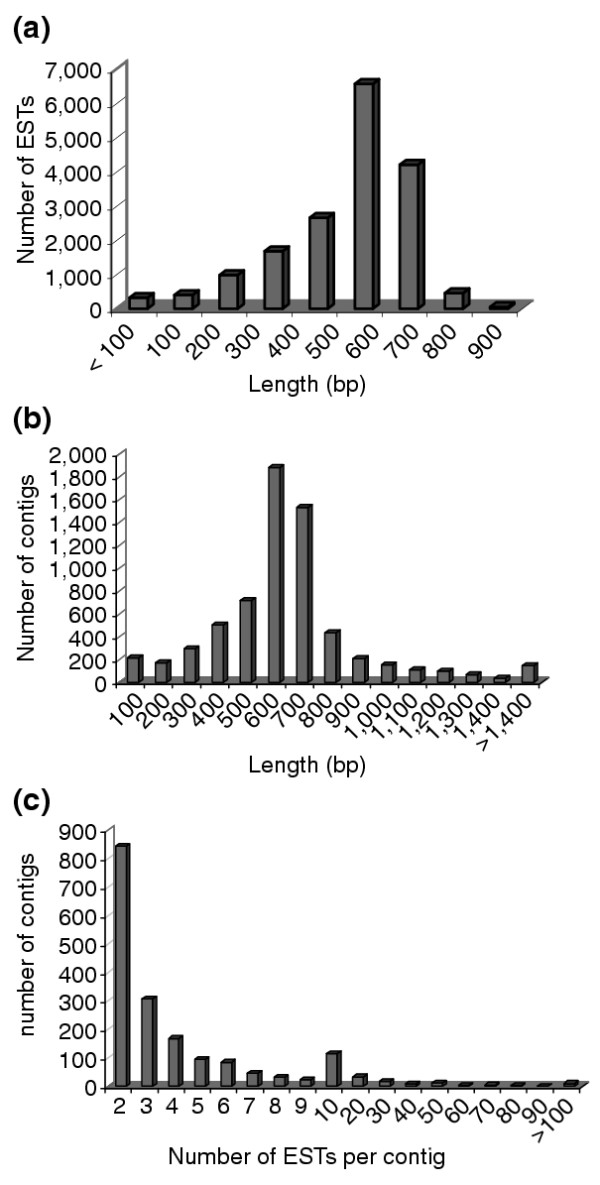
Distribution of sequence length. **(a) **Distribution of read lengths of the sequenced ESTs after quality control. The average read length was 569 bp, corresponding to a peak of between 500 and 600 bp. **(b) **Distribution of sequence length of assembled contigs. The average length of contigs was 597 bp. **(c) **Distribution of the number of ESTs per assembled contig. Most of the contigs had one EST. The two largest contigs contained over 400 ESTs (cytochrome *c *oxidase subunit I and 12S rRNA, respectively).

To generate a diverse set of sequences involved in organizing and regenerating the primary body axis, two independent cDNA libraries were used for sequencing. One was derived from dorsal regions of stage 18-22 embryos containing neural tube, somite and notochord - called the 'neural tube' library - the other from 6-day post-amputation regenerating tail blastema. From 18,432 sequencing attempts 17,522 high-quality sequences were obtained after Phred analysis [11]. All sequences are 5' reads of the inserts. Of 17,522 high-quality, single-pass sequencing runs, 32 clones contained no insert and 137 sequences were below 32 base pairs (bp). These sequences were excluded from further analysis (32 bp representing the lower limit for assembly of a sequence using TIGR-assembler), yielding 17,352 clones for final analysis. The neural tube library was the origin of 7,469 sequences and the blastema library of 9,883 sequences (Table 1, and see Materials and methods). As shown in Figure 1a, the average sequence read length peaked between 500 and 600 nucleotides with an average length of 510 nucleotides and a maximum of 871.

The blastema and neural tube libraries were unnormalized and unamplified. We assessed library quality and diversity on the basis of the number of redundant clones in the library. Redundancy was estimated by performing BLASTN searches [12] against all clones sequenced. After sequencing 10,752 clones of the blastema library 42% of the sequences were still unique, and 50% of clones were still singlets after sequencing 7,680 clones from neural tube, indicating that both libraries display high diversity.

### EST assembly into contigs

To identify ESTs belonging to the same open reading frames (ORFs), sequences were assembled into contigs using TIGR-Assembler version 2 [13]. The 17,353 sequences assembled into 6,594 contigs, of which 217 were less than 100 nucleotides long and excluded from further analysis. A total of 6,377 contigs was therefore left for final analysis (Table 1). Of these, 4,561 contigs contained a single clone. The average contig length of the remaining dataset was 616 nucleotides (Figure 1b). Other than singlets, most of the contigs consisted of two ESTs (884 contigs, Figure 1c). The largest contigs included cytochrome *c *oxidase subunit I (469 ESTs), 12S rRNA (445 ESTs), nuclear factor 7 Zn-binding protein A33 (332 ESTs), type II keratin (274 ESTs), keratin (211 ESTs) and cytoplasmic beta-actin (206 ESTs) (Table 2).

### Comparison to existing *A. mexicanum *genes in NCBI: 6,000 new contig sequences

A total of 1,134 ESTs were available from *A. mexicanum *in the National Center for Biological Information (NCBI) EST databases prior to this work, most of which originate from a sequencing effort of the Voss laboratory ([14] and S.R. Voss, D. King, N. Maness, J.J. Smith, M. Rondet, S.V. Bryant, D.M. Gardiner, and D.M. Parichy, unpublished work (NCBI-accession numbers BI817205-BI818091); see also [15]). We examined to what extent our EST dataset overlapped with the sequences available to date. Only 600 of the ESTs in the public database identified one of our contigs in a BLASTN search as a homolog; in 85% of cases, the E-value was below 1E-50 and the sequences can be considered as potentially identical. Existing ESTs in the database largely originate from regenerating limb (S.R. Voss, D. King, N. Maness, J.J. Smith, M. Rondet, S.V. Bryant, D.M. Gardiner and D.M. Parichy, unpublished work). There was, however, only a slight bias of matching contigs to regenerating blastema (49%) as compared to neural tube (44%). Seven percent of identified contigs were found in both libraries. These results mean that our EST data enriches the existing sequence resource of *A. mexicanum *with approximately 6,000 new gene sequences.

### BLAST analysis of *A. mexicanum *contigs to assign homologies

To identify putative homologies to known proteins, we subjected the contigs to BLASTX searches against the non-redundant protein database (NR, NCBI) where a cutoff E-value of 1e-05 was used for parsing output files. In our annotation, we used an E-value of 1e-20 as an upper limit to assign significant homology. We note that this does not imply that such sequences are true orthologs. In addition, in cases where no significant homology was found, we used an E-value limit of 1e-05 to designate weak homology. We find this additional category of 'weak homology' useful for data mining. As most contigs do not represent full-length sequences, it is possible that only a highly divergent region of a gene sequence is available in our collection. The category of weak homology allows us to find potential homologs in such situations. For example, the BLAST search for contig Am_4671 yielded the GenBank entry NP_004055, cyclin-dependent kinase inhibitor 1B (*Homo sapiens*), as the top hit with an E-value of 4e-07. This assignment was based on the carboxy-terminal 120 amino acids of the protein, which represents the less conserved region. When we isolated a full-length clone for Am_4671 from our library, we could confirm that it is indeed the axolotl ortholog of cyclin-dependent kinase inhibitor 1B (p27^Kip1^), as discussed later.

**Figure 2:**
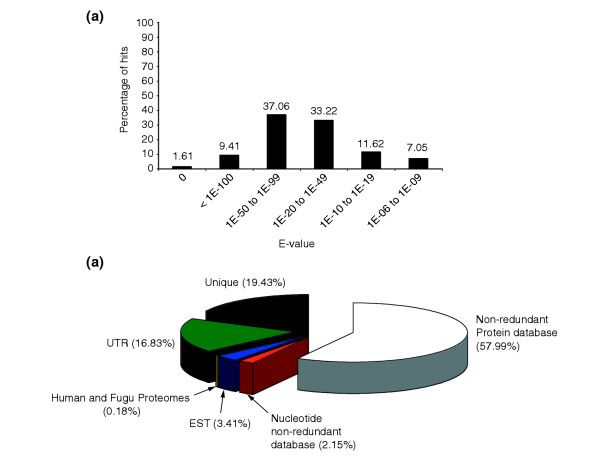
Homology of *A. mexicanum *contigs to protein and nucleotide sequences from other species. **(a) **Distribution of E-values from the first identified hit in the protein non-redundant database that was used to assign a putative identity to the contig. The majority of contigs identified a protein with an E-value between 1e-20 and 1e-99. In 11% of the cases, the E-value of the first hit was below 1e-100 and can therefore be considered a true ortholog. **(b) **Distribution of hits in the different sequence databases that were searched sequentially.

Taken together, a total of 3,718 (58%) sequences shared homology with a protein from selected model organisms in the non-redundant database and could be assigned a putative identity. The E-value distribution of the top hits in the non-redundant database is shown in Figure 2a. Of the contigs, 11% matched a protein with an E-value below 1e-99 and are therefore likely to be true orthologs. Seventy percent of the contigs found a hit with an E-value between 1e-20 and 1e-99 and were assigned significant homology. Finally, 19% of contigs had a first hit with an E-value between 1e-19 and 1e-05 and were assigned weak homology to a protein from the non-redundant database. For annotating our database, these top hits from human, mouse (*Mus musculus*), rat (*Rattus norvegicus*), frog (*X. laevis*), zebrafish (*Danio rerio*), fugu (*Takifugu rubripes*), fruitfly (*Drosophila melanogaster*), mosquito (*Anopheles gambiae*), worm (*Caenorhabditis elegans*), newts and the yeast species *Saccharomyces cerevisiae*, *Schizosaccharomyces pombe *and *Candida albicans *were collected and the closest homolog from the above species was used to assign a putative identity.

To estimate how many of the clones are full length we examined the BLAST alignments for the position of the alignment in respect to the database sequence. Of the 3,718 sequences with homologs, 1,107 (29.8%) could be aligned in the amino terminus (with the alignment starting before position 10). As the library was poly(dT) primed, many of these clones are likely to represent full-length inserts. Of these 199 (5.4%) could be aligned from the amino terminus to the carboxy terminus and are potential full-length sequences.

Forty percent of our EST sequences did not generate a significant hit in the non-redundant protein database. The availability of additional sequence databases including complete genome sequences from several organisms allowed us to expand our BLAST searches to identify all possible homologs to the *A. mexicanum *contigs. With the remaining set of contigs, we first performed BLASTN searches against the nucleotide non-redundant (NT) database and BLASTX searches against the EST database. Finally, we performed BLASTX searches against the fugu and human proteomes. In all cases, an E-value of 1e-05 was used to assign potentially homologous sequences. Sequences in the NT database identified an additional 134 contigs and a further 220 contigs found a hit in the EST databases. A homolog was found for 3,340 (52%) contigs in the fugu proteome and 3,698 (58%) contigs shared homology with a protein from the human proteome. In total, an additional 468 contigs identified a homolog in the selected databases beyond the original assignment from the non-redundant protein database (Figure 2b).

### Gene sequences with no identifiable homology

No homologous sequence could be found for 2,191 (34%) contigs in any of the databases searched. Because the library was poly(dT) primed, many of these sequences could represent 3' untranslated regions (3' UTRs). We determined that 953 sequences (43% of non-homologous contigs) contained no ORF and were therefore potential untranslated regions. Thirty of the sequences shared homology to an existing *A. mexicanum *clone from the EST database (Table 3). The complete list of unique ESTs can be downloaded from [16].

### Assignment of the *A. mexicanum *dataset to common Gene Ontology terms

From the homologous proteins found, contigs were assigned a biological process, molecular function and cellular component from the Gene Ontology (GO) database [17]. The closest annotated homolog in the GO database was used, using an E-value of 1e-20 as a cutoff, for assigning these categories. A biological process could be assigned to 2,156 contigs (34% of all contigs and 58% of those sharing a homolog in the non-redundant database); 2,186 contigs (34% and 59%, respectively) were assigned a molecular function; and 2,198 contigs (34% and 59%, respectively) could be assigned a cellular component. The most abundant molecular function assigned was 'death receptor interacting protein', followed by 'peptidase', the highest-ranking biological process were 'biological process unknown' and 'proteolysis/peptidolysis' and the most abundant cellular components assigned were the 'actin cytoskeleton' and 'transcriptional repressor complex'.

**Figure 3:**
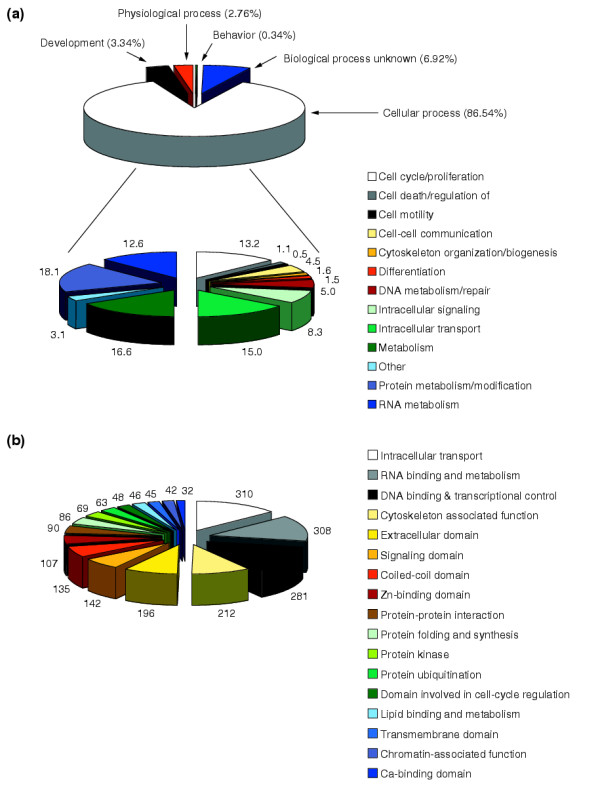
Annotated GO terms and protein domains in the *A. mexicanum *EST libraries. **(a) **Gene Ontology electronic annotation in the category 'biological process' of contigs from *A. mexicanum*. The largest proportion of annotated contigs was assigned a 'cellular process' (87%). Of those, five large groups of cellular processes emerged, with 'cell cycle/proliferation' (13%), 'intracellular signaling' and 'intracellular transport' (8% and 15%), 'metabolism' (17%), 'protein metabolism/modification' (18%) and 'RNA metabolism' (13%). **(b) **Domains associated with cellular processes identified in the *A. mexicanum *contig sequence dataset. The largest fraction of contigs was associated with a domain function in 'intracellular transport', followed by 'RNA-binding and metabolism' and 'DNA-binding and transcriptional control'.

The largest fraction of the contigs was assigned a cellular process in the GO category biological process (87% of annotated contigs) (Figure 3a). We split the biological processes further into different categories: the most abundant categories were 'protein metabolism/modification' (18% of assigned contigs); 'housekeeping functions/metabolism' (17%); 'intracellular transport' (15%); 'cell cycle/proliferation' (13%); 'RNA metabolism' (13%); 'intracellular signaling' (8%); and 'DNA metabolism/repair' (5%) (Figure 3a, Table 4). A list of annotated contigs is downloadable from [16].

### Common SMART and PFAM domains in the *A. mexicanum *dataset

To identify potential domains in the axolotl contigs, we performed RPS-BLAST searches against the conserved domain database (CDD, NCBI) [12,18] using the default cutoff E-value of 0.01. A total of 2,199 (34.5%) contigs had a known protein domain in either the CDD or the SMART or PFAM databases. A detailed list of common protein domains identified in our dataset is given in Table 5. Among the protein domains identified were homeobox domains such as HOX, PAX and Prox1, eight helix-loop-helix (HLH) domains, RNA-binding domains such as KH and RRM, 69 kinase domains, metal- and lipid binding domains and domains involved in cell-cycle control and ubiquitination (RING fingers, HECT domains, three cullin domains and 12 cyclin domains). Many of these domains were annotated for the first time in a sequence from *A. mexicanum*. We also compared the occurrence of those domains in other vertebrate species. For most of the common protein domains, only a fraction were found in our dataset; many of these are quite abundant compared to *X. laevis *or *Gallus gallus*. The RNA-binding domains KH and RRM especially showed high abundance in our contigs. A complete list of domains is downloadable from [16].

We assigned cellular functions to the identified domains and analyzed the output according to the functional distribution of contigs (Figure 3b). The most abundant domains were found in the category 'intracellular transport'; this is due to redundant annotations of small GTPases. The second largest fraction belonged to 'RNA-binding and metabolism', followed by 'DNA-binding and transcriptional control'.

### *In silico *differential display of *A. mexicanum *contigs in blastema and neural tube

#### Regeneration versus development

We were interested to see if there were strong differences in the sequence representation of the libraries that reflect the different biological processes taking place in each tissue. To this end, we compared the representation of ESTs in the two libraries. This type of *in silico *differential display has been performed for ESTs in the NCBI collection, and, as with the NCBI differential display data, we have assessed the statistical significance of the differences using Fisher's exact test. A total of 104 contigs met the cutoff value of 0.005 in Fisher's exact test and can therefore be considered differentially expressed.

Table 4 provides a detailed comparison of EST representation categorized according to their biological process annotation. Considering the biological properties of the blastema tissue versus the neural tube tissue, we were particularly interested in differential display results of gene sequences that had been assigned to the biological functions of RNA metabolism (as an indicator of an high proliferation index), cell cycle and proliferation and differentiation. The blastema library was produced from tail tissue that was in the process of forming the blastema progenitor cells for regeneration. Blastema formation involves dedifferentiation of mature cells, and entry into rapid cell cycles. In contrast, the neural tube library contains tissue undergoing cell specification and differentiation, such as neurogenesis and somitogenesis. Although these embryonic tissues are still proliferating, the proliferation index of the cells from neural tube should be lower than from blastema.

#### RNA metabolism

A total of 168 contigs annotated under RNA metabolism (127 when normalized to the ratio of sequenced ESTs from blastema and neural tube) were more frequently sequenced or uniquely sequenced in blastema (6% of assigned contigs, 2.6% of all contigs). This group included RNA metabolism, RNA processing, splicing, editing, nuclear export, binding, catabolism, cleavage, capping, rRNA modification, rRNA transcription and tRNA aminoacetylation. Forty-five contigs assigned a process in RNA metabolism were upregulated or unique in neural tube (2% of assigned and 0.7% of all contigs). After Fisher's exact test analysis, 24 of the clones were considered differentially regulated in the two libraries; 22 out of the 24 contigs were enriched or unique in blastema (Table 4).

#### Cell cycle and proliferation

126 contigs (95 when normalized to sequencing ratios) were assigned as cell-cycle genes (5% of assigned contigs and 1.5% of total contigs) and were more frequently sequenced or uniquely sequenced in the blastema library, compared with 52 in the neural tube library (2.5% and 0.8%, respectively). This category included regulation of mitosis, mitosis, cell-cycle regulation, regulation of cyclin-dependent kinase (CDK) activity, cell proliferation, DNA replication, M phase, mitotic spindle checkpoint, mitotic spindle assembly, chromosome segregation and cytokinesis. As an example, 10 different types of cyclins were found, from various stages of the cell cycle. Seven of the contigs found in cell-cycle regulation met the cutoff criteria of statistical significance in Fisher's exact test. Five out of the seven contigs were more highly represented or unique in blastema (Table 4).

#### Differentiation

Whereas proliferation-associated genes were found with a higher sequence representation in the blastema library, genes that had been electronically annotated as involved in 'cell differentiation' had a higher representation in the neural tube library. A total of 28 contigs were electronically assigned the biological process 'differentiation'. After Fisher's exact test, five contigs showed differential regulation in this group. Three out of the five contigs were found in neural tube (Table 4). Taken together, these results indicate that the two cDNA libraries have differences in sequence representation that appear to correlate with the physiological processes taking place in the two tissues.

### Gene families involved in cell-cycle control and development in the *A. mexicanum *dataset

As mentioned earlier, the Mexican axolotl is an important model organism for a number of reasons. First, it is the premier vertebrate model for studying regeneration. Second some aspects of caudate development, for instance mesoderm involution and notochord formation, more closely resemble those found in higher vertebrates than do those in other amphibian embryological models such as *X. laevis *[19]. Finally, the axolotl has interesting developmental features, particularly in relation to metamorphosis. The axolotl undergoes 'cryptic metamorphosis', which is defined by its existence in a perrenibranchiate state and retaining some larval features into adulthood (for instance gills, larval skin morphology, caudal fins). The animals become sexually mature in this state, and develop only small rudimentary lungs. So far, very few markers are available to study these processes in this organism.

We examined our dataset for genes that are potentially useful for studying regeneration features or developmental processes. To this end, we analyzed our data for genes that are either involved in regulating the cell cycle - as would be expected for the highly proliferative tissue of a regenerating body structure - or could play an essential role during development and metamorphosis from the larval to the adult stage. A list of genes that could be assigned to either cell-cycle regulation or development is shown in Table 6. Among the genes involved in cell-cycle regulation were A-, B- and E-type cyclins, cyclin-dependent kinase 4 (Cdk4), Polo kinase, the kinase inhibitor p27^Kip1^, the protein phosphatase Cdc25A, as well as the anaphase-promoting complex (APC) activator proteins Cdc20 and Cdh1. Representing genes involved in developmental processes, we found transcription factors such as HoxA2, B12, C4 and C8, Pax6, as well as Cdx1 and Cdx2. Furthermore we found several genes for proteins that are part of the transforming growth factor-beta (TGF-β) signaling pathway, such as TGF-β, bone morphogenetic protein 1 (BMP-1), BMP and activin membrane-bound inhibitor, activin receptor type II, as well as the transcription factors Smad5 and Smad8. Genes for proteins such as Smad8 and BMPs might be of especial interest to the research field of embryonic development, as they have been associated with mesoderm involution [20]. Other important developmental genes that could be found in our dataset include those for Wnt5 and Wnt8, Sonic hedgehog, retinoblastoma binding protein 2, beta-catenin, as well as Frizzled 2, 5 and 7. Finally, it has been shown that the thyroid hormone receptor pathway has an essential role in the timing of metamorphosis in *A. mexicanum *[21-23]. We identified the protein TRIP12 (thyroid hormone receptor interacting protein 12), which is a HECT-domain-containing ubiquitin ligase and could have an essential role in regulating thyroid hormone response during development and/or metamorphosis.

### Phylogenetic analysis of the CDKN1 gene family in vertebrates: amphibians contain an unusual CDKN1 family member

**Figure 4:**
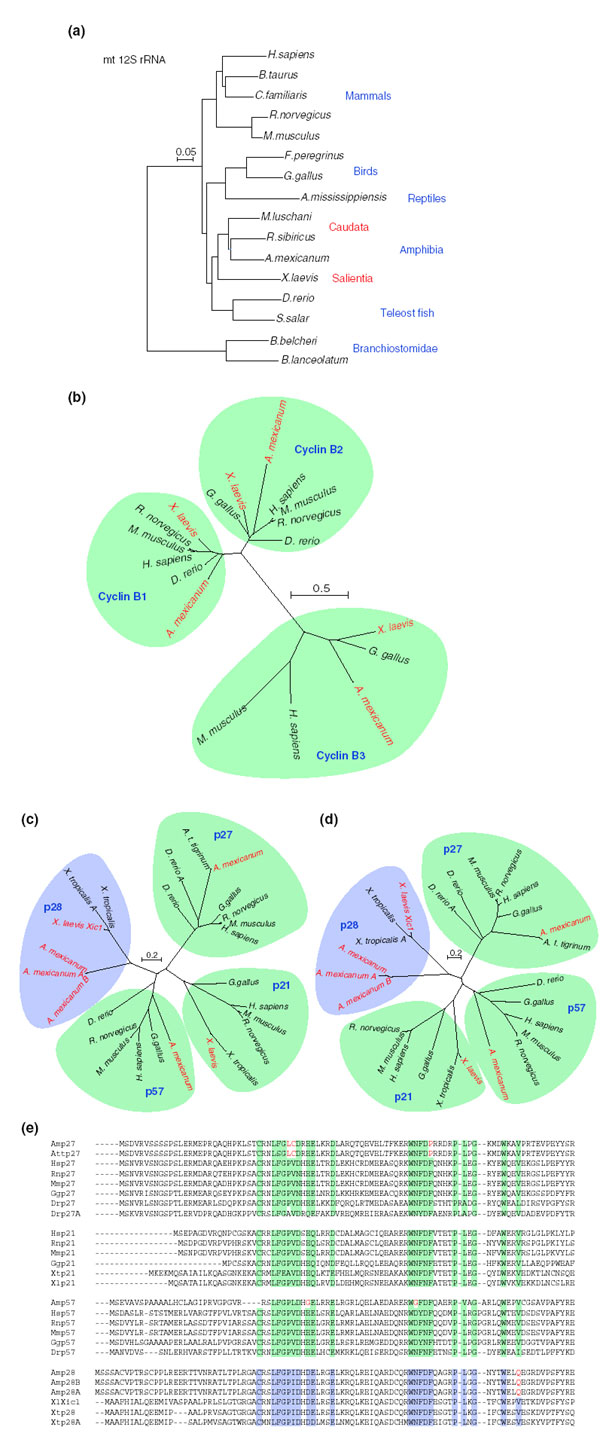
Phylogenetic analysis of the vertebrate cyclin-dependent kinase (CDK) inhibitors (CKIs) p21(Cip1), p27(Kip1) and p57(Kip2). **(a) **Reference phylogenetic tree of mitochondrial 12S rRNA. The Caudata and Salientia both branch out to build the amphibian group. **(b) **Unrooted phylogenetic tree of the cyclin B1 gene family. The amphibian cyclin B1 family members form a distinct group. **(c) **Unrooted phylogenetic tree of the amino-terminal CDK-inhibitory domain of vertebrate p21, p27, p28 and p57, which is conserved between the protein families. p27 of *A. mexicanum *clearly groups with the p27 proteins from other vertebrates. The amphibian-specific p28-family does not parse with any singe group. Note, however, that unlike the 12S rRNA tree, the *A. mexicanum *and *A. t. tigrinum *p27 branch out with that of *D. rerio*. **(d) **Unrooted, phylogenetic tree of the full-length kinase inhibitor sequences. Using the full-length protein sequences from the CKI families, the p28 family branches off between the p21 and p27 families. **(e) **Multiple sequence alignment of the amino-terminal, CDK-inhibitory region of the CKI families. The protein sequence of *A. mexicanum *p27 is clearly the ortholog of the p27 family, yet displays higher than expected divergence on the protein level. The same divergence is observed for the ambystomatid p57 proteins. The p28 family has extremely high sequence divergence compared to any other CDKN1 family member. Conserved residues between the three CDKN1 families are highlighted in green and the p28-family in light blue. Residues that differ between ambystomatid sequences and the other vertebrate species are highlighted in the ambystomatid sequences in red. Accession numbers are: NM_131513 (*D. rerio *ccnb1), NM_031966 (*H. sapiens *ccnb1), BC041302 (*X. laevis *ccnb1), NM_172301 (*M. musculus *ccnb1), NM_171991 (*R. norvegicus *ccnb1), P13351 (*X. leavis *ccnb2), XP_343420 (*R. norvegicus *ccnb2), P29332 (*G. gallus *ccnb2), NP_004692 (*H. sapiens *ccnb2), NP_031656 (*M. musculus *ccnb2), CAC24491 (*X. laevis *ccnb3), P39963 (*G. gallus *ccnb3), CAC94915 (*H. sapiens *ccnb3), NP_898836 (*M. musculus *ccnb3), AAH56746.1 (*D. rerio *p27A, Drp27A); AAK84219.1 (*D. rerio *p27, Drp27); CN056871.1 (*A. t. tigrinum *p27, Attp27); AAM22491.1 (*G. gallus *p27, Ggp27); NP_004055.1 (*H. sapiens *p27, Hsp27); P46414 (*M. musculus *p27, Mmp27); NP_113950.1 (*R. norvegicus *p27, Rnp27); NP_000067.1 (*H. sapiens *p57, Hsp57); P49919 (*M. musculus *p57, Mmp57); XP_341967.1 (*R. norvegicus *p57, Rnp57); CN039016.1 (*A. mexicanum *p57, Amp57); BM489375.1 (*G. gallus *p57, Ggp57); CK697132.1 (*D. rerio *p57, Drp57); AAH01935.1 (*H. sapiens *p21, Hsp21); NP_031695.1 (*M. musculus *p21, Mmp21); NP_542960.1 (*R. norvegicus *p21, Rnp21); AL639561.2 (*X. tropicalis *p21, Xtp21); BJ065460.1 (*X. laevis *p21, Xlp21); AAN63876.1 (*G. gallus *p21, Ggp21); I51683 (*X. laevis *Xic1, XlXic1); BX712320.1 (*X. tropicalis *p28, Xtp28); TNeu143i03.p1cSP6 (*X. tropicalis *p28A, Xtp28A); CN033557.1 (*A. mexicanum *p28, Amp28); CN035131.1 (*A. mexicanum *p28A, Amp28A); CN033708.1 (*A. mexicanum *p28B, Amp28B). The scale bar indicates substitutions per site.

The EST collection will provide rich data for the phylogenetic comparison of particular genes. Cell cycle and cell differentiation are cellular functions that have been modified in various organisms through evolution and it will be interesting to understand the evolutionary basis of such changes. Here we analyze a particularly interesting gene family, the CDKN1 family of cell-cycle regulators which inhibit cell-cycle progression by binding to and inactivating CDKs. As a starting point for phylogenetic analysis, the mitochondrial 12S ribosomal RNA gene from our collection resulted in the expected tree, with the anuran amphibian *X. laevis *and the caudate *A. mexicanum *grouping together compared to other vertebrates such as fish, birds and mammals (Figure 4a). Next, we constructed an unrooted phylogenetic tree to compare members of the cyclin B family - cyclins B1, B2 and B3. The sequences of each family member formed strictly separate groups, with the *A. mexicanum *and *X. laevis *cyclin B1, B2 and B3 genes grouping with their vertebrate orthologs (Figure 4b).

In contrast, we obtained a quite different picture when we examined the CDKN1 family. In most vertebrates, this family consists of three members: p21 (CDKN1A), p27^Kip1 ^(CDKN1B) and p57 (CDKN1C). In *X. laevis*, however, only a single family member called p28^Kix1 ^(also called p27^Xic1^), which shows unusual sequence features compared to the p27 sequences from any other vertebrate species, had been described in the literature [24,25]. We wondered whether *A. mexicanum *harbored the 'canonical' p27^Kip1 ^or a p28^Kix1 ^similar to that of *Xenopus*. We initially searched our *A. mexicanum *data for CDKN1 orthologs and, in contrast to *Xenopus*, we found a *bona fide *p27^Kip1 ^sequence that clusters closer to vertebrate p27^Kip1 ^sequences compared to the *Xenopus *p28^Kix1 ^(Figure 4c,d). Considering this interesting finding, we then undertook a more complete analysis of the CDKN1 family in vertebrates by searching for CDKN1 family members in several databases: the sequenced genomes from human, mouse, rat, fugu or zebrafish, the recently released genome sequence of *X. tropicalis*, the *X. laevis *EST collection, the zebrafish and fugu genomes, and a complementary *A. mexicanum *and *A. tigrinum *EST set generated by Putta *et al*. [26].

This data mining revealed two striking features about the distribution of CDKN1 family members among vertebrates (Table 7). First, the p28^Kix1 ^orthologs were only found in amphibians (*X. tropicalis*, *X. laevis*, *A. mexicanum*, *A. tigrinum tigrinum*). We were not able to identify a p28^Kix1^-like gene in any other database. These p28 orthologs group as a distinct branch in an unrooted phylogenetic tree (Figure 4c,d). These data so far suggest that the p28 family is a CDK inhibitor that is specific for amphibians. With new genome sequence data being released, it will be interesting to see whether the most closely related lineage of birds contains a p28-like gene or whether this gene family is found solely in amphibians.

Second, CDKN1B (p27^Kip1^) and CDKN1C (p57) were present in the *A. mexicanum *databases but were not found in either *X. laevis *or *X. tropicalis*, which have far more EST and genome sequence information (Table 7, Figure 4c,d). While it is not possible to conclude definitively that *Xenopus *species lack these genes, the current data are highly suggestive of such a scenario.

We examined in depth the phylogenetic relationships of the CDKN1 family members among vertebrates by constructing unrooted phylogenetic trees, either using the most conserved, amino-terminal 88-amino-acid domain, which includes the functionally important Cdk2-interaction region, or the entire coding sequence. Analysis of the amino terminus showed that while *A. mexicanum *p27 and p57 clearly grouped with their respective orthologs from other vertebrates, the p28^Kix1 ^proteins from axolotl and the two *Xenopus *species clustered as a group distinct from any of the other CDKN1 families (Figure 4c). The p28^Kix1 ^family showed a closer relationship to p57 than to other CDKN1 members, branching off close to the p57 family. Phylogenetic analysis using the entire coding sequence of the CDKN1 genes, which includes the Cdk2- and PCNA-binding site, resulted in a closer grouping of p28 with the p27 branch (Figure 4d). In both cases, however, the p28 family clearly formed a separate group from the other CDKN1 families.

### The *Ambystoma mexicanum *EST database

**Figure 5:**
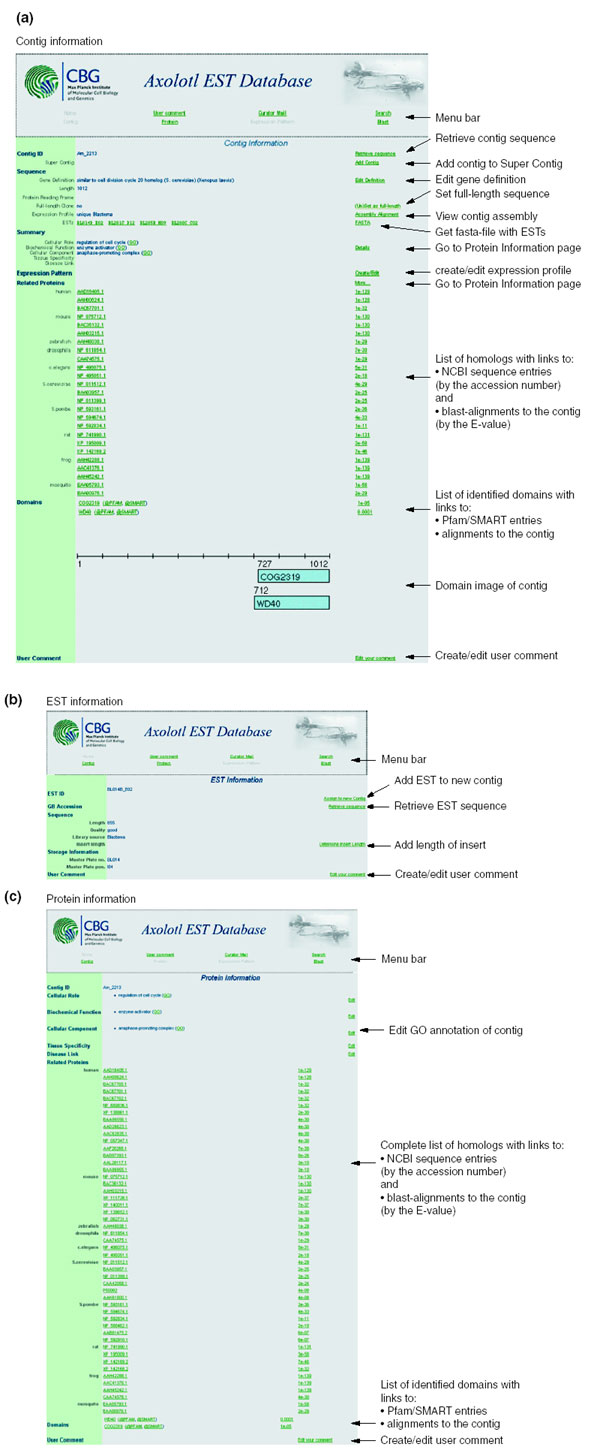
The *Ambystoma mexicanum *EST database. A relational database was created as a sequence storage and annotation resource of the sequenced ESTs from *A. mexicanum*. **(a) **The main entry site of the EST resource is the contig page, where a subset of the information is available, including the identity of included ESTs, putative identity of the contig, GO annotation including cellular role, biochemical function and cellular component, a list of homologs from different model organisms, and identified conserved domains. Source data are available for all BLAST-based alignments, for external sequence or domain data, and for the complete contig sequence. **(b,c) **EST information and protein information pages, containing more detailed description of storage information, library source and read length (b). A complete list of homologs and identified conserved domains can be assessed on the protein information page (c). For a more detailed description of the database, see text.

A relational database with a web-based front end was created to store, navigate and annotate analyzed contigs. The main object of the database is the annotated sequence contig, which contains information about its length, putative identity, computationally calculated expression profile, GO annotation, homologous proteins and identified domains, as well as number and identity of ESTs that build the contig (Figure 5a). The Gene Identifier (GI) and GO annotation can be modified by the administrator. To circumvent the problem of split contigs, we introduced a super-contig, to which related contigs can be assigned. Furthermore, the administrator can modify the relationship of EST to contig manually. All protein and domain alignments, as well as the assembly of the EST sequences of a contig are stored and can be viewed by the user. On the contig main page, three homologs at most from selected species are shown, with a full list of homologs from selected species displayed on the protein information page (Figure 5c). To make use easier, an image of the identified domains with the beginning and end base pair of the alignment is shown on the contig page. Individual ESTs can be accessed via the contig page, including their length, storage information, quality information and available trimmed EST-sequence (Figure 5b).

Some of the main advantages of this database are: first, the direct links to source databases such as the NCBI sequence database, GO database, CDD, and the Smart and Pfam databases for identified domains; second, direct visualization of source data such as sequence alignments of contigs to homologs and domains, as well as alignments of EST assemblies; third, easy retrieval of sequences for further analysis like BLAST-searching; fourth, user-specific annotation of contigs; and fifth, easy manipulation and editing of contig annotations. The database will be available from [27].

## Discussion

The salamander, and in particular the species *A. mexicanum, *represents an important vertebrate organism for evolutionary, developmental and regeneration studies. The salamanders provide an essential amphibian counterpoint to the anurans such as *X. laevis*, displaying distinct embryology and other physiological features. For example, mesoderm involution during gastrulation and subsequent notochord formation is distinctive between *A. mexicanum *and *X. laevis*. The characteristics of mesoderm involution in *A. mexicanum *more closely resemble those found in other vertebrates [19]. This and other evidence indicates that *A. mexicanum *and other urodele amphibians are likely to have retained more ancestral features in common with the 'primitive' tetrapod compared to *X. laevis*, which appears to be more derived. It is interesting that we observed such segregation on the sequence level of the CDKN1 family. *X. laevis *appears to have a highly unusual make-up of CDKN1 family members. So far, CDKN1A (p21) and the highly derived p28^Kix1 ^are the only CDKN1 family members found in both *X. laevis *and *X. tropicalis*. In contrast, the ambystomatids appear to have all the members of the CDKN1-family - including p28^Kix1 ^- assuming that the p21 gene is missing purely as a result of lack of sequence information. In addition, our data suggest that p28 is an amphibian-specific variant of the CDKN1 family. Two major questions arise from these data: first, does the amphibian-specific p28 fulfill a cellular function that is unique to this phylogenetic lineage; and second, does the genotypic difference in the gene set of the CDKN1 family in the two amphibian species account for the macroscopic differences observed in developmental mechanisms. The fact that the CDKN1 family is an essential regulator of the cell cycle opens new possibilities for experimental research along these lines.

Given the estimates in the number of genes present in the human genome (20,000-50,000) [28], we estimate that our EST contig set (6,377) contains between 10 to 25% of the total number of genes in the axolotl. While the database is not yet complete, it represents a significant proportion of the axolotl transcriptome. Further sequencing efforts, including an NIH-funded EST sequencing project for the axolotl [26], will enlarge the current dataset to provide a comprehensive gene sequence resource for this organism. Our analysis indicates that the majority of *A. mexicanum *genes are homologous to genes present in other vertebrates. Sixty-six percent of contigs gave a significant match in either the non-redundant protein or nucleotide databases, the EST databases or the human and fugu protein databases. Thirty-four percent of contigs could not be assigned a homolog in any of the searched databases, and 44% of those could not be assigned a coding sequence and are therefore considered to be part of the UTR. Nineteen percent of the contigs seem to represent novel genes that have not been found in any other organism so far.

The expressed sequence tags generated in this study also provide a large source of sequence information for developmental and regeneration studies. For example, an examination of the database yielded 194 genes involved in cell proliferation, including pivotal cell-cycle genes such as those for Cdc2, 10 different cyclin family members, Cdk4 and p27. A search for developmental molecules involved in intercellular communication yielded Wnt8, Wnt5B, FGF receptor 4a (FGFR4a), Sonic hedgehog, BMP receptor (BMPR) and BMP-1, while a search for homeodomain-containing proteins yielded 11 members, including Cdx1, Cdx2, HoxA2, HoxC8 and HoxB13.

The ESTs were derived from two cDNA libraries, stage 18-22 embryonic neural tube/notochord/somite tissue, and day-6 regenerating tail tissue. The embryonic library represents a developmental stage where tissue specification is occurring, whereas the blastema library represents a tissue that is undergoing dedifferentiation, rapid proliferation and cell respecification. Accordingly, we find differences in transcript representation in the two libraries. The blastema library is particularly enriched in cell-cycle genes and RNA metabolism genes, presumably reflecting the high proliferative index of the early regenerating blastema.

## Conclusions

This set of 17,352 ESTs from *A. mexicanum *was generated to provide a comprehensive sequence dataset for the community of biologists. Forty percent of genes could still be found in singlets, which reflects a high diversity of sequences in our cDNA set. Annotation of the assembled contigs revealed a substantial difference in gene representation in the two sequence libraries, reflecting their biological source - regenerating blastema being in a highly proliferative state and embryonic neural tube being a tissue undergoing differentiation. Sequence analysis of assembled contigs revealed that 64% of genes had a putative homolog in other species; 19.4% of the contigs contained a putative coding sequence and can be considered novel genes. From this, we conclude that *A. mexicanum *does not contain an unusually high number of organism-specific genes. The CDK inhibitor family CDKN1 was selected for comparative phylogenetic analysis. Unlike the frogs *X. laevis *and *X. tropicalis*, ambystomatids most probably contain all members of the CDKN1 family, including the amphibian-specific protein p28^Kix1^/p27^Xic1^, which shows unusual sequence divergence compared to CDKN1 members in other vertebrate species. Such data would support the contention that *A. mexicanum *is closer to a basal tetrapod compared to *X. laevis*. The EST sequences and annotated contigs presented in this paper will be a publicly available and useful resource for research in various fields.

## Materials and methods

### Plasmid cDNA library construction

Total RNA was purified using Trizol (Invitrogen) from 6-day regenerating tail blastemas and from neural tube-somite-notochord-containing tissue dissected from stage 18-22 *A. mexicanum *embryos. Total RNA quality was assessed by determining the relative brightness of the 28S:18S rRNA bands (2:1). For library construction mRNA was purified and size fractionated, then poly(dT)-primed cDNA was synthesized and directionally cloned into the *Not*I-*Sal*I sites of the pCMVSport6 vector. DNA was transformed by electroporation into EMDH10B-TONA bacteria (library construction performed by Invitrogen). Two separate, unnormalized libraries were produced. The blastema library contained an average insert size of 1.67 kb and 2.67 × 10^7 ^independent transformants and the neural tube library had an average insert size of 1.5 kb and 1.9 × 10^7 ^transformants. From each library 100,000 clones were arrayed into 384-well plates (Resource Zentrum/Primary Database, Berlin, Germany).

### Sequencing

For sequencing, single-pass reads from the 5' end of the library inserts were performed using a custom-designed SP6 primer: GCACATTAGGCCTATTTAGGTGACA. DNA from bacterial library clones was amplified using the Templiphi reaction, based on φ29 rolling-circle replication of DNA (AP Biotech). Briefly, approximately 0.5 μl of bacterial glycerol stocks were picked up using 96-pin plastic replicators (Genetix) and centrifuged into 96-well PCR plates. Five microliters of denaturing buffer was added, and samples heated to 95°C for 3 min. After cooling, 5 μl Templiphi enzyme was added and samples incubated overnight in a 30°C incubator. The Templiphi reaction provides two advantages for large-scale sequencing projects on capillary sequencers. First, the reaction proceeds to an endpoint where all nucleotide is incorporated, yielding uniform quantities of DNA from varying amounts of starting bacteria (or DNA). Second, the rolling-circle reaction results in large pieces of DNA that, in contrast to plasmid DNA, do not enter the capillary and interfere with the sequencing run.

For sequencing reactions, the DNA preparation was diluted fivefold with distilled water. Sequencing reactions were performed using the DYEnamic ET Dye terminator kit diluted twofold with DYEnamic ET dilution buffer (AP Biotech). Five microliters of DNA was added to 5 μl of sequencing reaction mix with primer and cycled 30 times under the following conditions: 95°C 20 sec, 60°C 1 min. Sequencing was performed on a MegaBACE 1000 (AP Biotech). Runs were either performed at injection: 3 kV 60 sec, run: 8 kV 120 min, or injection: 3 kV 60 sec injection, run: 3 kV 360 min.

### Analysis of library quality

The redundancy of the arrayed libraries was tested by performing BLASTN searches [12] against all sequenced ESTs from the two libraries. Hits against clones other than the query with an E-value lower than 1e-50 were considered for clustering.

### Submission of ESTs to NCBI GenBank

The sequences were submitted to GenBank. After quality control, individual ESTs were used to search the non-redundant protein database (release of July 2004) using the program BLASTX from the standalone NCBI-BLAST package [12]. For annotation of sequenced ESTs, the top hit of the BLAST output was used, whereby an E-value of 1e-20 was used for significant similarity and an E-value of 1e-05 was used as a cutoff value for weak similarity.

### Analysis and assembly of sequence data

Quality control of sequenced ESTs was performed using the program Phred [11] using a cutoff of 20 for trimming low-quality regions, and vector trimming was performed using the program cross-match [11]. (We note here that the arbitrary Phred score reflects the likelihood of a false base. A Phred score of 20 indicates that in 1 out of 100 trials (10^2^), the base would be false, 30 would reflect a wrongly sequenced base in 1 of 1,000 trials (10^3^), and so forth.) Sequence and contig files can be downloaded at [16]. The resulting high-quality sequences were assembled into sequence contigs with the program TIGR-Assembler version 2 [13]. Alignment of contigs was performed with the program ClustalW with the settings Gap Opening 5 and Gap Extension 85 [29] or Cap3 [30], when ClustalW could not correctly assemble the sequences. Assembled contigs were used to perform BLAST searches (BLASTX, BLASTN from NCBI-BLAST [12]) against the non-redundant protein sequence database (release of November 2003), human and fugu protein databases and the NCBI EST database, all downloaded from the NCBI. Domain searches were done with RPS-BLAST against the conserved domain database (CDD [18]) from the NCBI. BLAST and domain-search output files were parsed for homologous sequences, whereby an E-value of 1e-05 was used as a cutoff for BLASTN and BLASTX searches against the sequence databases and the default cut-off of 0.01 was considered to yield significant homology to conserved domains from CDD. A gene identifier was assigned to those contigs that showed reliable homology to a sequence in the non-redundant database (E-value cutoff of 1e-20 for significant similarity and 1e-05 for weak similarity). Potential untranslated regions were identified using the program ESTScan [31].

### Electronic annotation of contigs

Based on the GO annotation of the closest annotated homolog, contigs were assigned a molecular function, biological process and cellular component from the GO database [17]. To this end, the GenBank annotation files from the GO database were downloaded and parsed for the gene identifier (gi) numbers of previously identified homologs. The cutoff for annotating an *A. mexicanum *contig was an E-value of 1e-20.

### Isolation of the full-length p27^Kip1 ^gene from the EST sequence

Two EST sequences of the p27^Kip1 ^gene were sequenced in the EST collection but neither were full-length sequences. To isolate the full-length sequence, 200,000 clones of our arrayed blastema and neural tube libraries were screened by PCR. Briefly, the bacterial library clones in each 384-well plate were pooled, mini-prepped and arrayed into 96-well plates (RZPD, Berlin), resulting in 576 DNA pools. These DNA pools were screened by PCR using the custom SP6 primer (GCACATTAGGCCTATTTAGGTGACA) as a forward primer and a gene-specific p27 reverse primer (TGATTTCCAATGGCTGGTTT). Fifty nanograms of DNA from each pool was used for PCR reactions and PCR cycling was performed at the following conditions: 94°C 2 min, 30 cycles of 94°C 15 sec, 65.5°C 30 sec, 72°C 90 sec, followed by 72°C 7 min). The largest positive band (1.1 kb) was gel purified and sequenced on an ABI377 machine using the SP6 primer.

### Phylogenetic analysis

Multiple sequence alignments were done with the program ClustalX [32] using standard parameters. Phylogenetic analysis of mitochondrial 12S rRNA was done using the programs dnadist, phylogenetic analysis of the cyclin B family and the CDK inhibitor family (CKI family) was done using protdist, both from the Phylip package [33]. Trees were calculated with the program fitch from the same software package, using 100 iterations. For the CKI family, only the amino-terminal, CDK-inhibitory domain or the full-length sequences were used for construction of a phylogenetic tree. For the cyclin-B family, only the region overlapping in *A. mexicanum *contigs was used for tree construction. Trees were displayed using the program nj-plot [34] for the mitochondrial 12S rRNA tree and unrooted [34] for the CKI- and cyclin B families.

### Database design

A relational database was created using the open source software MySQL as the database server to store and navigate through resulting sequence contigs and annotations. Scripts connecting the web-based front end to the database were written in the programming language Python.

## Figures and Tables

**Table 1:** Some characteristics of the *A. mexicanum *EST contigs

Library	Number of sequences	Number of contigs (+ singlets)	Number of clones in contigs	Number of clones in singlets
St18-22 neural tube	7,469			
6D tail blastema	9,883			
Combined total	17,352	6,377	12,791	4,561

The number of expressed sequence tags sequenced from the two libraries blastema and neural tube, as well as the number of contigs, the number of clones in contigs and the number of clones found in singlets is shown.

**Table 2:** Gene definition of the most abundant contigs in the *A. mexicanum *EST libraries

Gene definition	Number of clones in contig
Cytochrome *c *oxidase subunit I	469
12S rRNA	445
Nuclear factor 7	332
Keratin type II	274
Keratin	211
Cytoplasmic β-actin	206

The gene with the highest number of clones identified was cytochrome *c *oxidase subunit I (469 clones in contig), followed by 12S rRNA (445) and nuclear factor 7 (332 clones in contig).

**Table 3:** Contig identities and GenBank identifiers of ESTs unique to *A. mexicanum*

Contig ID	GenBank identifier	UTR
Am_1065	BI817418.1	
Am_13	BI817561.1	
Am_1868	BI817299.1	
Am_1879	BI817273.1	UTR
Am_1986	BI817397.1	
Am_2156	BI817699.1	UTR
Am_2280	BI817354.1	
Am_242	BI817917.1	
Am_2631	BI817344.1	
	BI818040.1	
	BI817371.1	
Am_2695	BI818066.1	UTR
Am_2767	BI817941.1	UTR
Am_2952	BI817736.1	
Am_3070	BI817303.1	
Am_3486	BI817478.1	
Am_3807	BI817992.1	UTR
Am_3828	BI817981.1	
	BI817250.1	
Am_4598	BI817704.1	
Am_4661	BI817548.1	UTR
Am_4720	BI817653.1	UTR
Am_5031	BI817804.1	UTR
Am_5579	BI818004.1	
Am_5650	BI817315.1	
Am_5742	BI817525.1	UTR
Am_5881	BI818060.1	
Am_6107	BI817553.1	UTR
Am_6128	BI817667.1	UTR
Am_6198	BI817866.1	
Am_646	BI817520.1	
	BI817607.1	
	BI817743.1	
Am_6565	BI817313.1	UTR
Am_901	BI817984.1	

The table shows contig identities and GenBank identifiers of existing *A. mexicanum *ESTs that do not share any homology to a known protein or nucleotide sequence and can therefore be considered unique.

**Table 4:** The most abundant biological processes assigned to the *A. mexicanum *contigs

Biological process	Total number of contigs	% contigs	BL/NT	Fisher's exact (BL/NT)
Protein metabolism	324	15	116/132	3/1
Metabolism	296	13.7	78/170	0/3
Intracellular transport	268	12.4	59/53	4/5
RNA metabolism	227	10.5	127/45	22/2
Cell cycle	194	9	95/52	5/2
Intracellular signaling	148	6.8	95/65	1/6
DNA metabolism/repair	90	4.1	50/12	3/0
Development	69	3.2	32/27	0/2
Cell-cell communication	81	3.7	24/42	0/6
Differentiation	27	1.5	13/7	2/3

The highest-ranking biological process is 'protein metabolism/modification' with 15% of contigs assigned. 'Cellular metabolism', 'intracellular transport' and 'RNA metabolism' have all more than 10% of contigs assigned and represent the most abundant gene families in the two libraries. The percentage contigs refers to the number of contigs assigned a biological process. BL: Blastema; NT: Neural tube.

**Table 5:** Common protein domains identified in the *A. mexicanum *contigs and comparison to domain occurrences in other vertebrate species

Domain	*A. mexicanum*	*H. sapiens*	*M. musculus*	*X. laevis*	*G. gallus*	*D. rerio*
EF-hand	10	319	308	36	48	38
Cyclin	12	60	58	20	9	15
Chromo	5	26	26	8	5	5
Prox1	5	4	2	2	1	2
HLH	8 (1)	167	179	83	70	75
HOX	13 (19)	280	352	196	142	250
PAX	1 (4)	12	31	25	9	13
EGF	10	310	281	26	50	32
SET	2	82	64	4	3	1
RAS	37	220	194	34	11	27
RhoGEF	4	124	98	4	2	3
PH	2	453	374	14	10	18
PX	4	70	74	2	0	3
WD40	39	547	490	63	12	50
Cullin	3	8	20	0	0	2
F-box	2	119	130	11	0	8
HectC	3	64	66	4	2	1
RING	17	374	325	18	16	29
KH	23 (1)	71	52	20	7	10
RRM	101 (2)	443	438	94	23	69
PDZ	8	260	252	17	11	23
Kinase	69 (2)	949	954	210	122	156
LIM	5	128	125	22	19	22
PHD	4	164	122	13	4	3

Numbers in parentheses indicate the number of domains that had been annotated to a protein sequence from *A. mexicanum *prior to this project.

**Table 6:** Gene families identified that are either involved in cell-cycle control or developmental processes

Cellular process	Putative ID of contig	Contig	Expression
Cell cycle	Cyclin A2	Am_20	BL unique
	Cyclin B1	Am_1031	BL 3x
	Cyclin B2	Am_4185	NT unique
	Cyclin B3	Am_3173	BL unique
	Cyclin E1	Am_38	BL unique
	Cyclin E2	Am_91	BL unique
	Cdk4	Am_3891	BL unique
	Polo kinase	Am_1717	BL unique
	Cdc25A	Am_3678	BL unique
	p27/Kip1	Am_4671	NT unique
	Cdc20	Am_2213	BL unique
	Cdh1	Am_1148	BL unique

Development	Wnt8	Am_384	BL unique
	Wnt5	Am_642	BL unique
	FGFR4a	Am_1393	BL unique
	Sonic hedgehog	Am_3741	BL unique
	Activin receptor type II	Am_3590	BL unique
	TGF-β	Am_4990	NT unique
	BMP-1	Am_4639	NT unique
	Cdx1	Am_875	BL unique
	Cdx2	Am_387	BL unique
	HoxA2	Am_2387	BL unique
	HoxB13	Am_4865	NT unique
	HoxC4	Am_3998	BL unique
	HoxC8	Am_2910	BL unique
	Pax6	Am_2945	BL unique
	Smad5	Am_1420	BL unique
	Smad8	Am_4665	NT unique
	Retinoblastoma binding protein 2	Am_2723	BL unique
	Beta-catenin	Am_699	BL 3x
	Zic5	Am_2068	BL unique
	Frizzled 2	Am_3243	BL unique
	Frizzled 5	Am_3451	BL unique
	Frizzled 7	Am_2334	BL unique
	TRIP12	Am_6416	NT unique

The identifier of the *A. mexicanum *contig is given in the third column. The expression pattern as determined by *in silico *differential display is shown in column 4.

**Table 7:** Occurrence of CKI-family members in different vertebrate species

	Human	Zebrafish	Fugu	*Xenopus tropicalis*	*Ambystoma mexicanum*
CDKN1A (p21)	+	-*	+	+	-*
CDKN1B (p27^Kip1^)	+	+	+	-^†^	+
CDKN1C (p57)	+	+	+	-^†^	+
p28^Kix1^	-	-	-	+^‡^	+^‡^

* Genes most likely present, yet not identified due to limited sequence information; † genes not present in genomic sequence information; ‡ genes so far only present in amphibian species. Databases searched were the human, mouse, rat, fugu, zebrafish and *X. tropicalis *genome databases, and the EST databases for *X. laevis*, *X. tropicalis*, zebrafish, *A. mexicanum *and *A. tigrinum*.
